# Clinical standards for the management of adverse effects during treatment for TB

**DOI:** 10.5588/ijtld.23.0078

**Published:** 2023-07-01

**Authors:** K. P. Singh, A. C. C. Carvalho, R. Centis, L. D’Ambrosio, G. B. Migliori, S. G. Mpagama, B. C. Nguyen, R. E. Aarnoutse, A. Aleksa, R. van Altena, P. K. Bhavani, M. S. Bolhuis, S. Borisov, N. van't Boveneind-Vrubleuskaya, J. Bruchfeld, J. A. Caminero, I. Carvalho, J. G. Cho, L. Davies Forsman, M. Dedicoat, K. Dheda, K. Dooley, J. Furin, J. M. García-García, A. Garcia-Prats, A. C. Hesseling, S. K. Heysell, Y. Hu, H. Y. Kim, S. Manga, B. J. Marais, I. Margineanu, A-G. Märtson, M. Munoz Torrico, H. M. Nataprawira, E. Nunes, C. W. M. Ong, R. Otto-Knapp, D. J. Palmero, C. A. Peloquin, A. Rendon, D. Rossato Silva, R. Ruslami, A. M. I. Saktiawati, P. Santoso, H. S. Schaaf, B. Seaworth, U. S. H. Simonsson, R. Singla, A. Skrahina, I. Solovic, S. Srivastava, S. L. Stocker, M. G. G. Sturkenboom, E. M. Svensson, M. Tadolini, T. A. Thomas, S. Tiberi, J. Trubiano, Z. F. Udwadia, A. R. Verhage, D. H. Vu, O. W. Akkerman, J. W. C. Alffenaar, J. T. Denholm

**Affiliations:** 1 Department of Infectious diseases, University of Melbourne, at the Peter Doherty Institute for Infection and Immunity, Melbourne, VIC, Australia; 2 Victorian Infectious Disease Unit, Royal Melbourne Hospital, VIC, Australia; 3 Laboratório de Inovações em Terapias, Ensino e Bioprodutos (LITEB), Instituto Oswaldo Cruz, Fundação Oswaldo Cruz, Rio de Janeiro, RJ, Brazil; 4 Servizio di Epidemiologia Clinica delle Malattie Respiratorie, Istituti Clinici Scientifici Maugeri Istituto di Ricovero e Cura a Carattere Scientifico (IRCCS), Tradate, Italy; 5 Public Health Consulting Group, Lugano, Switzerland; 6 Kilimanjaro Christian Medical University College, Moshi, , United Republic of Tanzania; 7 Kibong’oto Infectious Diseases Hospital, Sanya Juu, Siha, Kilimanjaro, United Republic of Tanzania; 8 Woolcock Institute of Medical Research, Viet Nam and University of Sydney, NSW, Australia; 9 Department of Pharmacy, Research Institute for Medical Innovation, Radboud University Medical Center, Nijmegen, The Netherlands; 10 Grodno State Medical University, Grodno, Belarus; 11 Asian Harm Reduction Network (AHRN) and Medical Action Myanmar (MAM), Yangon, Myanmar; 12 Indian Council of Medical Research-National Institute for Research in Tuberculosis, University of Groningen, Groningen, the Netherlands; 13 Department of Clinical Pharmacy and Pharmacology, University Medical Center Groningen, University of Groningen, Groningen, the Netherlands; 14 Moscow Research and Clinical Center for Tuberculosis Control, Moscow, Russia; 15 Department of Public Health TB Control, Metropolitan Public Health Services, The Hague, The Netherlands; 16 Departement of Medicine Solna, Division of Infectious Diseases, Karolinska Institutet, Stokholm, Sweden; 17 Departement of Infectious Diseases, Karolinska University Hospital, Stockholm, Sweden; 18 Department of Pneumology. University General Hospital of Gran Canaria “Dr Negrin”, Las Palmas, Spain; 19 ALOSA (Active Learning over Sanitary Aspects) TB Academy, Spain; 20 Paediatric Department, Vila Nova de Gaia Hospital Centre, Vila Nova de Gaia Outpatient Tuberculosis Centre, Vila Nova de Gaia, Portugal; 21 Sydney Infecious Diseases Institute (Sydney ID), The University of Sydney, Sydney, NSW, Australia; 22 Westmead Hospital, Sydney, NSW, Australia; 23 Parramatta Chest Clinic, Parramatta, NSW, Australia; 24 School of Pharmacy, Faculty of Medicine and Health, The University of Sydney, Sydney, NSW, Australia; 25 Department of Infectious Diseases, Heartlands Hospital, University Hospitals Birmingham NHS Foundation Trust, Birmingham, UK; 26 Centre for Lung Infection and Immunity Unit, Department of Medicine, Division of Pulmonology and UCT Lung Institute, University of Cape Town, Cape Town, South Africa; 27 South African Medical Research Council Centre for the Study of Antimicrobial Resistance, University of Cape Town, Cape Town, South Africa; 28 Faculty of Infectious and Tropical Diseases, London School of Hygiene & Tropical Medicine, London, UK; 29 Division of Infectious Diseases, Department of Medicine, Vanderbilt University Medical Center, Nashville, TN, USA; 30 Department of Global Health and Social Medicine, Harvard Medical School, Boston, MA, USA; 31 Tuberculosis Research Programme, SEPAR (Sociedad Española de Neumología y Cirugía Torácica), Barcelona, Spain; 32 Desmond Tutu TB Centre, Department of Paediatrics and Child Health, Stellenbosch University, Tygerberg, South Africa; 33 Department of Pediatrics, University of Wisconsin, Madison, WI, USA; 34 Division of Infectious Diseases and International Health, University of Virginia, Charlottesville, VA, USA; 35 Department of Epidemiology, School of Public Health and Key Laboratory of Public Health Safety, Fudan University, Shanghai, China; 36 Tuberculosis Department Latin American Society of Thoracic Diseases, Lima, Peru; 37 Department of Infectious Diseases and Microbiology, The Children’s Hospital at Westmead, Westmead, NSW, Australia; 38 Centre of Excellence in Infectious Diseases Research, Antimicrobial Pharmacodynamics and Therapeutics Group, Department of Pharmacology and Therapeutics, University of Liverpool, Liverpool, UK; 39 Clínica de Tuberculosis, Instituto Nacional de Enfermedades Respiratorias, México City, Mexico; 40 Division of Paediatric Respirology, Department of Child Health, Faculty of Medicine, Universitas Padjadjaran, Hasan Sadikin Hospital, Bandung, Indonesia; 41 Department of Pulmonology of Central Hospital of Maputo, Maputo, Mozambique; 42 Faculty of Medicine of Eduardo Mondlane University, Maputo, Mozambique; 43 Infectious Disease Translational Research Programme, Department of Medicine, Yong Loo Lin School of Medicine, National University of Singapore, Singapore,; 44 National University of Singapore Institute for Health Innovation & Technology (iHealthtech), Singapore; 45 Division of Infectious Diseases, Department of Medicine, National University Hospital, Singapore; 46 German Central Committee Against Tuberculosis (DZK), Berlin, Germany; 47 Hospital Muniz and Instituto Vaccarezza, Buenos Aires, Argentina; 48 Infectious Disease Pharmacokinetics Laboratory, College of Pharmacy and Emerging Pathogens Institute, University of Florida, Gainesville, FL, USA; 49 Universidad Autonoma de Nuevo Leon, Facultad de Medicina, Neumología, CIPTIR, Monterrey, Mexico; 50 Faculdade de Medicina, Universidade Federal do Rio Grande do Sul, Porto Alegre, RS, Brazil; 51 TB/HIV Research Centre, Faculty of Medicine, Universitas Padjadjaran, Bandung, Indonesia; 52 Department of Biomedical Sciences, Division of Pharmacology and Therapy, Faculty of Medicine, Universitas Padjadjaran, Bandung, Indonesia; 53 Department of Internal Medicine, Faculty of Medicine, Public Health, and Nursing, Universitas Gadjah Mada, Yogyakarta, Indonesia; 54 Centre for Tropical Medicine, Faculty of Medicine, Public Health and Nursing, Universitas Gadjah Mada, Yogyakarta, Indonesia; 55 Division of Respirology and Critical Care, Department of Internal Medicine, Faculty of Medicine, Universitas Padjadjaran/Hasan Sadikin General Hospital, Bandung, Indonesia; 56 University of Texas Health Science Center at Tyler, Tyler, TX, USA; 57 Department of Pharmaceutical Biosciences, Uppsala University, Uppsala, Sweden; 58 Department of TB & Respiratory Diseases, National Institute of TB & Respiratory Diseases, New Delhi, India; 59 Republican Research and Practical Centre for Pulmonology and Tuberculosis, Minsk, Belarus; 60 National Institute of Tuberculosis, Lung Diseases and Thoracic Surgery, Faculty of Health, Catholic University, Ružomberok, Vyšné Hágy, Slovakia; 61 Department of Medicine, The University of Texas at Tyler School of Medicine, TX, USA; 62 Department of Pharmacy Practice, Texas Tech University Health Science Center, Dallas, TX, USA; 63 Department of Clinical Pharmacology and Toxicology, St Vincent’s Hospital, Sydney, NSW, Australia; 64 Department of Pharmacy, Uppsala University, Uppsala, Sweden; 65 Infectious Diseases Unit, IRCCS Azienda Ospedaliero-Universitaria di Bologna, Policlinico di Sant’Orsola, Bologna, Italy; 66 Department of Medical and Surgical Sciences, Alma Mater Studiorum University of Bologna, Bologna, Italy; 67 Blizard Institute, Barts and The London School of Medicine and Dentistry, Queen Mary University of London, London, UK; 68 Department of Infectious Diseases, Austin Hospital, Melbourne, VIC, Australia; 69 P. D. Hinduja National Hospital and Medical Research Centre, Mumbai, India; 70 Department of Paediatrics, University Medical Center Groningen, University of Groningen, Groningen, the Netherlands; 72 Department of Pulmonary Diseases and Tuberculosis, Groningen, University of Groningen, Haren, the Netherlands; 73 Tuberculosis Center Beatrixoord, University Medical Center Groningen, University of Groningen, Haren, the Netherlands; 74 Victorian Tuberculosis Program, Melbourne Health, VIC, Australia

**Keywords:** tuberculosis, adverse effects, management, toxicity, drugs, safety

## Abstract

**BACKGROUND:**

Adverse effects (AE) to TB treatment cause morbidity, mortality and treatment interruption. The aim of these clinical standards is to encourage best practise for the diagnosis and management of AE.

**METHODS:**

65/81 invited experts participated in a Delphi process using a 5-point Likert scale to score draft standards.

**RESULTS:**

We identified eight clinical standards. Each person commencing treatment for TB should: Standard 1, be counselled regarding AE before and during treatment; Standard 2, be evaluated for factors that might increase AE risk with regular review to actively identify and manage these; Standard 3, when AE occur, carefully assessed and possible allergic or hypersensitivity reactions considered; Standard 4, receive appropriate care to minimise morbidity and mortality associated with AE; Standard 5, be restarted on TB drugs after a serious AE according to a standardised protocol that includes active drug safety monitoring. In addition: Standard 6, healthcare workers should be trained on AE including how to counsel people undertaking TB treatment, as well as active AE monitoring and management; Standard 7, there should be active AE monitoring and reporting for all new TB drugs and regimens; and Standard 8, knowledge gaps identified from active AE monitoring should be systematically addressed through clinical research.

**CONCLUSION:**

These standards provide a person-centred, consensus-based approach to minimise the impact of AE during TB treatment.

## Abstract (fr)

**CONTEXTE:**

Les effets indésirables (AE) du traitement de la TB sont une cause de morbidité, de mortalité et d’interruption du traitement. L’objectif de ces normes cliniques est d’encourager une meilleure pratique en matière de diagnostic et de prise en charge des AE.

**MÉTHODES:**

Ont participé 65/81 experts invités à un processus Delphi utilisant une échelle de Likert en 5 points pour évaluer des ébauches de normes.

**RÉSULTATS:**

Nous avons identifié huit normes cliniques. Chaque personne commençant un traitement antituberculeux devrait : Norme 1, être informée des AE avant et pendant le traitement ; Norme 2, être évaluée afin de détecter tout facteur susceptible d’augmenter le risque d’AE et faire l’objet d’un examen régulier afin d’identifier et de prendre en charge ces facteurs de manière proactive ; Norme 3, en cas d’AE, être évaluée avec soin et tenir compte d’éventuelles réactions allergiques ou d’hypersensibilité ; Norme 4, recevoir des soins appropriés pour minimiser la morbidité et la mortalité associées aux AE ; Norme 5, reprendre les médicaments antituberculeux après un AE grave selon un protocole standardisé avec une surveillance active de l’innocuité des médicaments ; Norme 6, les agents de santé doivent être formés aux AE, y compris à la manière de conseiller les personnes qui suivent un traitement antituberculeux, ainsi qu’à la surveillance et à la prise en charge actives des AE ; Norme 7 : tous les nouveaux médicaments et schémas antituberculeux doivent faire l’objet d’une surveillance active des AE et d’une notification ; et Norme 8 : les lacunes en matière de connaissances identifiées grâce à la surveillance active des AE doivent être systématiquement comblées par la recherche clinique.

**CONCLUSION:**

Ces normes fournissent une approche centrée sur la personne et fondée sur le consensus afin de minimiser l’impact des AE pendant le traitement de la TB.

TB disease requires treatment with a combination of drugs over several months.^1^–^3^ Adverse effects (AE) are associated with poor treatment adherence, treatment interruption and treatment failure.^4^–^8^ Early identification and management of AE is essential to avoid the development of resistance or disease progression. In some cases, the drug and or regimen may need to be permanently discontinued and alternative drugs used. AE occur in 25–75% of people receiving treatment for TB.^9^–^11^ The incidence of AE varies across studies due to heterogeneity in definitions of an AE, reporting thresholds/criteria for defining causality, study design,^12^,^13^ population characteristics (including concomitant medications), medication formulations and administration regimens. When new symptoms or laboratory abnormalities appear during treatment, differentiation is needed between AE, a new disease process, or a paradoxical reaction (e.g., immune reconstitution inflammatory syndrome [IRIS]). When AE are thought likely to be related to TB drugs, the use of combination therapy may make it difficult to ascertain which drug in a regimen is responsible. There is limited evidence for strategies to manage AE during treatment for TB. The clinical standards presented here are based on expert opinion and the best available evidence available. New evidence will be considered as it emerges with periodic future updates planned. The manuscript also highlights key research needs for optimising AE management during the treatment of drug susceptible (DS-) and drug-resistant (DR-) TB.

The IJTLD Clinical Standards for Lung Health complement existing WHO or other guidelines and integrate their recommendations to focus on person-centred care. The Standards are broad principles formulated with the understanding that they may have to be adapted and contextualised due to legal, organisational or economic reasons in diverse settings. We acknowledge differences in capacity but hope that these clinical standards will help to shape service development and facilitate access to additional resources to improve treatment outcomes for people with TB.

## AIM OF THE CLINICAL STANDARDS

This consensus-based document describes the following standards:

Each person commencing treatment for TB should:

1. Be counselled regarding AE before and during treatment for TB.
2. Be evaluated for factors that might increase AE risk with regular review to actively identify and manage these.
3. When AE occur, be carefully assessed and possible allergic or hypersensitivity reactions considered.
4. Receive appropriate care to minimise the morbidity and mortality associated with AE.
5. Be restarted on TB drugs after a serious AE according to a standardised protocol that includes active drug safety monitoring (aDSM);
  In addition:
6. Healthcare workers (HCW) should be trained on AE including how to counsel people undertaking TB treatment, as well as active AE monitoring and management.
7. There should be active AE monitoring and reporting for all new TB drugs and regimens.
8. Knowledge gaps identified from active AE monitoring should be systematically addressed through clinical research.

Some elements relate to previously published standards^14^,^15^ and these are highlighted when relevant (e.g., therapeutic drug monitoring is discussed in detail in ‘Clinical standards for the dosing and management of TB drugs’^14^).

## METHODS

A panel of 81 global experts representing the main scientific societies, associations and groups active in TB was identified, and invited to participate. Two declined and 14 did not respond. The 65 respondents included TB clinicians (*n* = 37), TB public health (*n* = 4), TB paediatricians (*n* = 8), TB pharmacologists (*n* = 13), a methodologist (*n* = 1), an immunologist (*n* = 1) and an epidemiologist (*n* = 1). Respondents were asked to comment using a Delphi process on an initial draft including eight standards developed by a core coordination team. A 5-point Likert scale was used (5: high agreement; 1: low agreement). At the first Delphi round, agreement was high, with a median value of 5 for all eight standards. Based on substantial agreement on the standards, a draft document was jointly developed by the coordination team. After two rounds of revisions, the document was approved by consensus (100% agreement).

## STANDARD 1

### Each person commencing treatment for TB should be counselled regarding AE before and during treatment for TB

Providing education and counselling to all people diagnosed with TB before and during their treatment is a core component of national and international treatment guidelines.^15^–^17^ Education and counselling should be person-centred and tailored appropriately considering the person’s current health, health literacy, language, culture and planned treatment regimen^18^,^19^ Clinicians should consider including people from the person’s support network (e.g., nominated family or friends). Health education and counselling is intended to empower people with TB to understand the disease, the benefits, and risks of the treatment and the importance of treatment adherence, emphasising the benefits of optimal treatment to both the person with TB and their family and community. Education and counselling should cover the importance of regular clinic assessment for early detection of AE, non-harmful drug effects (e.g. red/orange discoloration of body fluids by rifampicin,^20^ how to deal with symptoms including when and how to contact the treating team to report these and seek medical attention. Education and counselling should also include circumstances in which self-cessation of drugs should occur pending review, such as signs and symptoms of potentially life-threatening and/or irreversible AE (e.g., hepatotoxicity, visual impairment, or severe cutaneous AE). Educational materials about AE should be developed internationally and adapted locally involving stakeholders such as HCW (e.g., nurses, treating physicians, pharmacists), people affected by TB and their caregivers. Materials should be person-centred, include information about AE (as outlined in the previous paragraph) as well as general information about TB, the importance of treatment, adherence, and disease prevention. Digital resources may improve access to information and communication between HCW and people undergoing treatment for TB, including regarding AE.^21^

## STANDARD 2

### Each person commencing treatment for TB should be evaluated for factors that might increase AE risk with regular review to actively identify and manage these

Demographic, medical, social and behavioural characteristics have been associated with increased risk of AE during treatment for TB (Table 1).^14^ The association with increased risk may be related to factors such as age, genetics or alcohol use on drug pharmacokinetics. Other factors may cause morbidity or toxicity that overlap with AE, such as other causes of hepatitis and peripheral neuropathy (e.g., diabetes, substance use disorder). Pre-treatment clinical assessment should include evaluation for these risk factors. Identification of a risk factor should prompt clinicians to optimise management (e.g., nutrition, control of diabetes) and to follow up more closely/frequently for actively monitoring. Baseline laboratory testing (before starting treatment) may identify additional risk factors and provide a reference to enable recognition of new changes that occur during treatment. All people with TB should undergo testing for HIV. Other recommended baseline tests if feasible and/or clinically indicated include liver function tests (LFT), kidney function (serum creatinine) and complete blood count and pregnancy test if relevant. Tests for hepatitis B virus (HBV) and hepatitis C virus (HCV) should be performed if there are epidemiologic risk factors or baseline LFT derangement.

**Table 1:** Risk factors for the development of AE during treatment for TB.

Risk factor	Comments and references
Older age	Associated with hepatotoxicity^27^,^62^,^63^ May be due to changes in body composition, reduced enzymatic activity and renal insufficiency^43^
Family/personal history of AE, atopy or allergy	Associated with cutaneous AE and hypersensitivity^64^
HIV	Data are mixed and systematic evidence is lacking, but trends for increased AE among people living with HIV, especially hepatotoxicity and hypersensitivity reactions have been described^1^,^65^–^67^ Cotrimoxazole may play a role in toxicity^68^ Associated with hypothyroidism in children prescribed ETH and PAS^69^
Viral hepatitis	Associated with increased hepatotoxicity^70^–^72^
Substance use disorders	Alcohol use was associated with higher risks of treatment interruption and unfavourable outcome^7^,^73^ Smoking in combination with alcohol may further increase risk^74^,^75^
DM	Associated with four-fold or more increase in AE^76^ Increased risk of peripheral neuropathy/ocular neuropathy^13^,^77^ In MDR-TB treatment, increased nephrotoxicity and hypothyroidism risk (older regimens)^73^,^78^.
Renal impairment	Associated with ethambutol and CS^13^/terizidone, aminoglycoside and fluoroquinolone toxicity due to increased drug concentrations^73^,^79^.
Pregnancy, early post-partum	Increased risk of hepatotoxicity, neuropathy^80^
Low weight/BMI	Variable association of low weight with increased rates of AE,^81^ especially hepatotoxicity^82^
Malnutrition, hypoalbuminemia, anaemia	Malnutrition associated with increased risk of hepatotoxicity^83^–^86^ Interferes with drug clearance which can lead to accumulation of drugs in the body and AE Anaemia was an independent risk factor associated with AE^87^,^88^ Hypoalbuminemia was an independent risk factor for TB drug-induced hepatotoxicity,^89^ including in children^90^
More advanced TB	Extrapulmonary TB in either people with TB or TB-HIV was an independent risk factor associated with AE^91^
MDR-TB	Higher rates of AE have been reported compared to treatment of DS-TB but many based on older regimens^49^, ^60^ Relatively higher incidence of AE leading to medication discontinuation associated with use of injectable agents, CPM, PAS and also linezolid (lower risk with FQ, BDQ, CFZ)^92^
Concomitant medications/polypharmacy	Drug interactions and overlapping AEs may be important Common in individuals with co-infections or dual disease, e.g., TB-HIV or TB-DM^93^–^96^ Pharmacokinetics and/or pharmacodynamic drug–drug interactions may result in overlapping adverse drug reactions or synergy in the occurrence of AE^97^,^98^

AE = adverse event(s); ETH = ethionamide; PAS = para-aminosalicylic acid; CS = cycloserine; DM = diabetes mellitus; MDR-TB = multidrug resistant TB; BMI = body mass index; DS-TB = drug-susceptible TB; CPM = capreomycin; BDQ = bedaquiline; CFZ = clofazimine.

The effect of pharmacogenomics (i.e., the way an individual’s genetic makeup affects the response to therapeutic drugs) is more difficult to assess. Variations (polymorphisms) in the *N*-acetyltransferase 2 (*NAT2*) gene, responsible for the metabolism of isoniazid was significantly associated with the likelihood of experiencing TB drug related hepatotoxicity.^22^ A randomised controlled trial demonstrated that the risk of drug-induced liver injury due to isoniazid could be reduced in adult slow acetylators by a dose reduction to 2.5 mg/kg/day without early treatment failure.^23^

In some cases where an AE is unable to be reliably detected or reported, an alternative agent may need to be substituted. For example, in the case of diminished visual acuity or colour blindness the clinician may decide not to use ethambutol (EMB) and/or linezolid, due to the as risk of optic neuropathy.

For all persons with TB, active surveillance for AE should occur according to a defined framework, including systematic and structured clinical assessment. As per Standard 1, pre-treatment counselling and education regarding when and how to access attention for symptoms that may appear during treatment and that may indicate the development of AE should be reinforced. The development of service structures that enables timely access to HCW review is also important. Assessment of new symptoms should always include consideration of and assessment for other causes, e.g., viral hepatitis in the case of hepatitis. Periodic laboratory testing may help to identify early AE, but cost-benefit assessments have not been done.^24^,^25^ Repeat laboratory testing may be warranted during the intensive phase of treatment (when most AE occur) and is important if baseline tests were abnormal, in the case of risk factors (e.g., underlying liver or renal/kidney disease, concomitant medications), pregnancy/early post-partum, ongoing treatment with pyrazinamide (beyond intensive phase) or at any time in the case of presumed AE (Table 2).

**Table 2:** Assessment and monitoring for AE using active drug safety monitoring ^*^ .

	Targeted clinical assessment	Tests	Additional assessments only if second-line drugs are used
Before treatment	Presence of risk factors (see Table 1) Extent of TB disease/dissemination Pregnancy/breast feeding	Colour vision,^†^ visual acuity Pregnancy (urinary/serum HCG) Mental health/neuropsychiatric assessment^‡^ HIV LFT (ALT), creatinine, CBC Hepatitis B/C serology HbA1c	If QT prolonging agents (FQs, CFZ, BDQ, DLM), measure electrolytes (K^+^, Ca^++^, Mg^++^) and ECG If DLM, measure albumin If ETH or PAS, measure TSH If aminoglycoside/CPM (now uncommon): assess hearing and bedside vestibular function. Formal pure tone audiometry for high frequency detection is optimal.
During treatment	Gastrointestinal upset Hepatotoxicity (e.g., anorexia, nausea, vomiting, fever, fatigue, pruritis, jaundice (icterus), abdominal discomfort, easy bruising/bleeding, hepatomegaly Joint pain/arthritis (gout) Neuropathy (e.g., tingling, numbness, burning hands or feet), altered gait, refusal to walk Blurry or altered vision (optic neuropathy) Rash; characterise nature and location/extent (e.g., mucosal involvement), severity, systemic features (fever, nausea) Pregnancy/breast feeding Neuropsychiatric disorders, including depression, anxiety, hallucinations, delusions – assess severity, presence of suicidal ideation Anaemia (tiredness, pallor shortness of breath)	Symptoms should prompt further clinical assessment and relevant testing (e.g., colour vision^†^/visual acuity, mental health assessment, LFT) Laboratory tests without symptoms or baseline abnormalities may not be needed unless risk factors are present (e.g., LFT in the presence of viral hepatitis or underlying cirrhosis) Consider tests for other causes, e.g., LFT derangement, test for hepatitis A (especially children)^99^ Rash, if severe, assess for organ dysfunction: LFT/creatinine, eosinophils (DRESS syndrome)	If QT prolonging agents (e.g., FQs, BDQ, CFZ, DLM), measure repeat electrolytes (serum K^+^, Ca^++^, Mg^++^) and ECG (QT interval) at 2w, 12w, 24w (or in case there are suggestive symptoms, e.g., dizziness, fainting, palpitations, syncope) If LZD, measure monthly CBC If ETH or PAS, monitor for hypothyroidism (every 2 months, additionally if clinically indicated) If FQs, assess for arthralgia, arthritis, tendonitis; be aware of rare AEs, such as raised intracranial pressure (headache) If CFZ, warn those undergoing treatment; monitor for skin discolouration, ichthyosis If aminoglycoside/CPM (now rare), assess regularly (monthly, 3/6 months after completion) for renal function and ototoxicity (hearing, vestibular dysfunction)

^*^ During treatment the frequency of review should be tailored to the individual person, treatment regimen/drugs used and the likely risk. Early during treatment and particularly during the intensive phase when most AEs are likely to occur review should be more frequent (e.g., every 2–4 weeks). During all phases additional review in case of new symptom development or concern is important.

^†^ For example, Ishihara testing to assess colour vision; charts are available online.^100^

^‡^ Mental health assessment tools that are accessible and assess the range of potential side effects across TB treatments are limited.^101^ Those that do exist may not be relevant and/or validated across populations (e.g., age, culture). Assessment should focus on AEs of the drug being used and use locally validated tools applicable to age.

AE = adverse effects; HCG = human chorionic gonadotropin; LFT = liver function test; ALT = alanine transaminase; CBC = complete blood count; HbA1c = glycated haemoglobin; FQ = fluoroquinolone; CFZ = clofazimine; BDQ = bedaquiline; DLM = delamanid; K^+^ = potassium; Ca^++^ = calcium; Mg^++^ = magnesium; ECG = electrocardiogram; ETH = ethionamide; PAS = para-aminosalicylic acid; TSH = thyroid stimulating hormone; LZD = linezolid; CPM = capreomycin; DRESS = drug reaction with eosinophilia and systemic symptoms; w = weeks.

In assessing AE, attention should be given to recognising reactions in which morbidity is likely and for which drug therapy may need to be withheld (Table 3). This will vary on a case-by-case basis depending on comorbidities and severity of TB disease. Use of first-line therapy for TB is associated with raised liver transaminases in about 20% of those treated, which spontaneously resolves over days to weeks without alteration in therapy.^26^ All individual agents have been associated with hepatotoxicity aside from EMB.^27^ Transaminase increases of over five times the upper normal limit (ULN) or three times the ULN in the presence of symptoms suggestive of hepatic toxicity (e.g., nausea, vomiting) should lead to therapy being withheld (Table 3).^27^ Treatment with rifamycins is associated with increased alkaline phosphatase and bilirubin concentrations without adverse consequences. Treatment should therefore continue if transaminases remain below these thresholds.^26^ In people with miliary/disseminated TB, increased transaminases are frequently observed. They may be present prior to and improve with treatment. Decisions regarding withholding treatment due to LFT derangement should consider these possibilities, as well as the potential for worsening with treatment due to paradoxical reactions.

**Table 3:** Identification and further assessment of ‘red flag’ symptoms (that may be associated with high risk of poor outcomes and necessitate withholding of medication), including criteria for identifying allergic/hypersensitivity reactions.

Criteria	Additional information needed	Possible reaction
Timing of onset <1 h after dose administration^*^	Nature of reaction: urticarial rash, pruritis, with systemic features, e.g., flushing, angioedema of face, extremities or laryngeal tissues (e.g., throat tightness or stridor), wheezing, gastrointestinal symptoms and/or hypotension	Type I hypersensitivity reaction (may occur with any drug)
Petechial rash	Platelet count	Suggests thrombocytopenia from a rifamycin hypersensitivity
Moderate–severe rash or other severe cutaneous reaction	Blistering, mucosal involvement, systemic features (fever, nausea/vomiting)	Severe cutaneous adverse event, e.g., Steven’s Johnson syndrome, drug reaction with eosinophilia and systemic symptoms (‘DRESS’) or toxic epidermal necrolysis (may represent Type IV hypersensitivity reaction). Most common with rifampicin
Loss of appetite, nausea, vomiting, abdominal pain, especially with jaundice	Examination for evidence of acute hepatitis (e.g., jaundice, RUQ tenderness) Liver tests (including transaminases, bilirubin, alkaline phosphatase)	Severe hepatotoxicity (AST/ALT > 5× ULN or >3× ULN with symptoms or underlying cirrhosis/liver disease) Pyrazinamide and INH are most commonly associated with hepatotoxicity (Standard 4)^102^
Visual change	Test colour vision (e.g., Ishihara plates) and visual acuity (e.g., Snellen chart), compare to baseline	Ocular toxicity including optic neuritis may be related to EMB, LZD or rarely INH
‘Fullness’ in ears, hearing loss and vertigo/nystagmus	Otoscopy: rule out wax Audiometry assessment if available	Aminoglycosides/capreomycin (or streptomycin) related ototoxicity
Neuropsychiatric adverse effects	Seizures	Seizures may be due to CS,^103^ FQs, LZD, INH, carbapenems; may also be symptoms of TB disease
	In case of depression, assess for severity – is suicidal ideation present?	CS^103^ and ETH (less common INH/EMB)
	Psychosis	CS, FQs (less commonly INH)
	In children: assess for hallucinations and night terrors	DLM, CS/terizidone, FQs
Renal failure		Rifamycins,^104^–^106^ aminoglycosides

^*^ Type I hypersensitivity reactions may be delayed if taken with food, with cases reported up to 6h after dosing.

DRESS = drug reaction with eosinophilia and systemic symptoms; RUQ = right upper quadrant; AST = aspartate transaminase; ALT = alanine aminotransferase; ULN = upper limit of normal (local range); EMB = ethambutol; LZD = linezolid; INH = isoniazid; CS = cycloserine; ETH = ethionamide; FQ = fluoroquinolone; DLM = delamanid

## STANDARD 3

### Each person commencing treatment for TB should, when AE occur, be carefully assessed and possible allergic or hypersensitivity reactions considered

A systematic approach is needed to assess possible AE, documenting the nature and severity and the likelihood that it is related to TB drugs. Tools such as the Naranjo Scale may be used to assess the likelihood of a causal relationship with drug administration.^28^ This scale includes the timing of onset and AE progression relative to drug administration, presence of other possible causes, including other prescription or over-the-counter medications, and whether there is objective confirmation of the AE (clinical assessment or laboratory testing). It is important to ascertain whether the AE has led to treatment modification (including self-initiated) or interruption and if so, the period and whether symptoms have changed. AE are most common soon after starting the drug; however, a time lag prior to their development (including allergic reactions) is also possible. More than 80% of AE are classified as ‘Type A’ reactions. Type A reactions are predictable from drug properties, may be recognised from previous reports and are sometimes referred to as ‘augmented reactions’. They are usually dose-related (Table 3). Type A reactions can usually be managed symptomatically or by modification of treatment times or doses (Table 4). In case of dose adjustment, therapeutic drug monitoring (TDM) is a valuable tool but is infrequently available.^14^ ‘Type B’ reactions are idiosyncratic (unpredictable) and may occur even after a sub-therapeutic dose. Type B reactions include hypersensitivity reactions (those mediated by an immunologic or inflammatory mechanism). Allergic reactions are those in which an immunologic mechanism has been demonstrated. Information on Type B reactions is mainly from case reports and a few case series, therefore evidence of the relative frequency of Type B reactions between different drugs is limited.

**Table 4:** How to support people when continuing TB therapy in the context of mild–moderate AE ^*^ .

Modality	Examples
Structural and timing	Change timing of doses (for sleep disturbance or daytime nausea) Split doses of medication (for pill burden or nausea) Positioning (e.g., sit upright after doses to avoid reflux)
Psychological	Contextualisation ○ “Can you tolerate the joint discomfort knowing that pyrazinamide will stop in 2 weeks?” ○ “In case you stop this drug, the treatment duration of other drugs will have to be prolonged?”) Reassurance (“The urine colour change is from your rifampicin, and isn’t harmful”) Education (“Your cough is likely caused by TB rather than your medication”)
Pharmacological	Analgesia (for joint pain) Anti-emetics (for nausea/vomiting), confirm that this is not caused by hepatotoxicity (LFT) Antihistamines (for itch, non-severe rash) Change of drugs within a class (e.g., moxifloxacin for levofloxacin) Supplemental levothyroxine if hypothyroidism due to TB drugs Management of peripheral neuropathy with (increased) vitamin B6 supplementation with INH (limited data)
Topical therapies	Moisturisers and/or sunscreen (for dry skin) Makeup or coloured skin products (for clofazimine discoloration) Anti-acne topical medication (for acne associated with INH use, especially the face)

^*^ The table lists examples of possible adverse effects and management options – it is not intended to be comprehensive or proscriptive.

LFT = liver function test; INH = isoniazid.

Additional tests for possible drug hypersensitivity such as skin prick, intradermal, patch or in vitro tests^29^ are used in some centres to further characterise reactions, but access to these tests and expertise on their use, is limited.^30^–^33^ Furthermore, research is needed to validate these tests and to establish non-irritant concentrations before they are more widely recommended. In vitro testing has also been explored,^34^ but is unproven and inaccessible in most settings.

## STANDARD 4

### Each person commencing treatment for TB should receive appropriate care to minimise the morbidity and mortality associated with AE

Every person undergoing treatment for TB should also be provided with appropriate care to manage AE as part of routine free-of-charge TB care. People experiencing AE should be supported with strategies that allow safe continuation of treatment when possible. This may include alterations in drug formulation^35^ (e.g., use of a dispersible or liquid) or administration (e.g., drugs taken at night rather than morning), as well as the use of ancillary therapies for symptomatic relief (Table 4). AE should be followed up to resolution or stabilisation as part of routine care.^36^ Additional therapies for symptomatic relief may include anti-emetics, steroids, antihistamines, analgesia or other medications, as well as non-pharmacological interventions (Table 4). When starting new drugs, including those administered to manage symptoms, always check drug–drug interactions, especially when using rifamycins. Before adding ancillary medication, care should also be taken to alert the patient to possible additional AE associated with their use (e.g., anti-emetics, antihistamines and several other medications prolong QT interval). In children, HCWs should be aware of the possibility of neurologic AE related to administration of anti-emetics (dystonia, which may be interpreted as convulsions). The entire treating team should be aware of any alterations that are made to the treatment regimen. Psychological support is an important element of managing any AE. Some AE may be mild or self-limiting, and psychological support alone may be sufficient to enable successful continuation of therapy. Aligning discussion to individually motivating factors is an important element of providing person-centred care.^37^

It is rarely appropriate to reduce doses of TB drugs in response to AE, as doing so may compromise effectiveness and increase the risk of drug resistance.^14^ Exceptions include where trial data have an established role, such as dose reduction of linezolid following onset of anaemia (myelosuppression) and cytopenia (Table 5).^38^,^39^ TDM may be useful, for example, to determine whether drug concentrations are supratherapeutic (possibly toxic) or when dose modification is used to manage symptoms of presumed AE.^14^ In case of reducing doses, drug susceptibility information (phenotypic and genotypic, when available) and microbiological monitoring can also be useful to monitor for disease relapse and selection of resistance. Lower doses of TB drugs may sometimes be used as part of an established protocol for drug escalation, such as for re-introduction in the setting of intolerance or desensitisation for some hypersensitivity reactions (Supplementary Data).

**Table 5:** Proposed protocol for rechallenge of TB drugs after specific AE.^1^,^3^,^27^,^29^,^32^,^41^

Adverse reaction	Probable causative drug	Rechallenge protocol
Cutaneous AE (sometimes known as cutaneous adverse drug reactions or ‘CADR’)	An individual drug can cause multiple types of cutaneous AE and a specific type of AE can be due to any drug Most reported:^*^ Drug hypersensitivity: INH, RIF, SM, PZA Stevens–Johnson syndrome; toxic epidermal necrolysis: RIF, PZA, INH, EMB, SM, CS, FQs	Rechallenge (drug provocation testing) is not recommended in severe cutaneous adverse events (e.g., Stevens-Johnson syndrome, toxic epidermal necrolysis) or immediate hypersensitivity. Rechallenge after rash has substantially improved, starting the drugs individually at intervals of 2–3 days, in the following order: RIF, followed by INH, then EMB or PZA. If rash reappears, the last drug added is withdrawn. In cases where three drugs were reintroduced without rash, the fourth drug may not be reintroduced unless the rash was mild and that drug essential In drug hypersensitivity, rapid desensitisation may be useful, see Supplementary Data for suggested protocol. For rapid desensitisation protocol, see Supplementary Data
Drug fever	INH RIF (Differential diagnosis: LZD (serotonin syndrome)	Body temperature is usually normal within 24 hours after drugs are withdrawn. Once afebrile, restart drugs as indicated for cutaneous AE
Optic/peripheral neuropathy	INH, LZD, CS^38^,^39^	Cease LZD INH/CS: vitamin B6 supplementation
Myelosuppression	LZD	Reduce LZD dose to 300 mg daily^39^
Hepatotoxicity	Most common with PZA and INH	Rechallenge if ALT levels <2× ULN, bilirubin normal and symptoms abated Start simultaneously with EMB and RIF, INH and PZA at usual doses, every 2–3 days Repeat LFT measurements before reintroducing a new drug If symptoms recur or ALT increases, the last drug reintroduced should be withdrawn Reintroduce full treatment over a period of up to 21 days^†^ If RIF, EMB and INH were reintroduced without further increases in transaminases, PZA can be suspended, in which case TB treatment should be extended to 9 months (continuation phase extended from 4 to 7 months)

^*^ Information on Type B reactions is mainly from case reports and a few case series, therefore evidence regarding the relative frequency of Type B reactions between different drugs is limited.

^†^ Time for reintroduction is uncertain. Minimising the time without effective therapy should be balanced against the risk/morbidity associated with AE-related toxicity.

AE = adverse effect; INH = isoniazid; RIF = rifampicin; SM = streptomycin; PZA = pyrazinamide; EMB = ethambutol; FQ = fluoroquinolone; LZD = linezolid; CS = cycloserine; ALT = alanine aminotransferase; ULN = upper limit of normal; LFT = liver function test.

In some cases, interruption or cessation of TB treatment will be needed to minimise morbidity, considering morbidity associated with TB that may result from delaying or inadequate treatment as well as morbidity associated with AE. In severe, miliary or central nervous system (CNS) TB, therapy may need to be continued using alternative (second-line) agents. Indications for interruption or cessation of TB treatment include ocular toxicity, renal failure, severe hepatotoxicity, hypersensitivity reactions and some neuropsychiatric disorders. Accurate diagnosis is important, especially in the case of hypersensitivity reactions, as this will help to determine which strategies are appropriate for further assessment and possibilities for continuing with therapy, and in a few cases, identifying AE that preclude restarting the drug (uncommon) or the need for alternative agents/regimens (see Standard 3).

New medications such as bedaquiline and pretomanid are effective and well-tolerated in treatment of DR-TB,^38^,^40^ and may be considered as second-line therapy in the case of AE during the treatment of DS-TB. Their tolerability and efficacy substantially improve options in the management of AE, although price and availability remain limitations to their use.

## STANDARD 5

### Each person commencing treatment for TB should be restarted on TB drugs after a serious AE according to a standardised protocol that includes active drug safety monitoring

AE related to TB drugs can often be managed with counselling and education without discontinuing medications. Cessation of drugs may be indicated with serious and/or worsening AE (Table 3). For hepatotoxicity, consensus thresholds for cessation have been established,^27^ while for many others assessment of severity and subjective evaluation of tolerability will guide timing of discontinuation. Optic neuritis, ototoxicity, severe acute nephrotoxicity, haemolytic anaemia, thrombocytopenic purpura, severe depression/suicidal ideation, psychosis, seizure or encephalopathy are examples of serious AE where treatment should be withheld and rechallenge may be contraindicated (see Table 3 for attributable TB drugs).^1^,^3^,^41^ Reintroduction of the standard regimen should be sought whenever possible. It is important that this is clearly explained to the person and their support network/caregivers. The decision on whether to reintroduce the ‘culprit’ drug will depend on the severity of the AE and risk of a more severe reaction after rechallenging, and on the severity of TB disease vs. the risk of a less potent and/or longer treatment regimen. Sequential reintroduction is used in some cases (drugs reintroduced one at a time) and should always start with the drug with the least probability to produce the AE, and to follow in order of probabilities.

Persons with severe forms of TB generally require initiation of an alternative regimen during the time the drug(s) presumed to induce toxicity are withheld and the rechallenge has not been completed.^1^,^3^,^41^ If the drug cannot be reintroduced, the person should be managed as if they had “functional resistance” to the drug.

### Drug-induced hepatotoxicity

Protocols for rechallenge of TB drugs are best described for cases of drug-induced hepatitis, although randomised controlled trials are needed to compare strategies for reintroducing drugs after hepatotoxicity. Most international guidelines^1^,^3^,^27^,^41^ follow the recommendation of reintroducing TB drugs after transaminase levels (especially alanine transaminase [ALT]) decrease to less than two times the ULN, bilirubin levels return to normal (for adults over 18 years old, normal total bilirubin is less than 1.2 mg/dL [20.5 micromol/L] of blood; in those aged <18 years, the upper limit is 1 mg/dL) and the person has no symptoms of hepatotoxicity. For those in whom there should be no treatment interruption (e.g., severe TB disease, CNS disease), three drugs not associated with hepatotoxicity may be administered while awaiting transaminase levels to decrease (Table 5). Sequential reintroduction of TB drugs at their usual doses should be done every 2–3 days (so that transaminase measurements can be repeated) over a period of no more than 7–10 days, generally in the following order: EMB, rifampicin (which can be reintroduced at the same time as EMB), isoniazid. If all other agents are tolerated, then continuing treatment without pyrazinamide is a reasonable strategy. If pyrazinamide is not included during the intensive phase of therapy, the continuation phase would need to be extended from 4 to 7 months.^3^,^13^,^42^,^43^ If reintroduction of the drug triggers a hepatocellular AE similar to or of greater severity than the previous one, this drug should no longer be used in future regimens for this person.^1^,^3^,^27^,^41^ Sequential rechallenge may allow determination of which medication is the cause of the hepatotoxicity; however, in a randomised trial that excluded individuals at increased risk of hepatotoxicity, no increased hepatotoxicity was observed when drugs were restarted concurrently.^44^,^45^ Some guidelines recommend a graded rechallenge (with incremental dosing increase; however, there is no evidence to suggest that this is better than full-dose rechallenge.^27^,^45^,^46^

### Cutaneous AE due to TB drugs

In case of significant cutaneous reactions in presumed drug hypersensitivity (Table 3), all TB drugs should be stopped. Mild cutaneous reactions, such as small surface area urticaria, may permit continued therapy and observation with antihistamines if symptoms are tolerable. Judicious drug provocation testing may be used to exclude drugs deemed unlikely to cause the reaction by restarting. Sequential testing in inverse order of probability of causation (starting with the drug least likely to cause the reaction based on currently available evidence) is recommended. Drug provocation testing is undertaken where the risk/probability of a hypersensitivity reaction (HSR) to a drug is thought to be low, with the intention of demonstrating clinical tolerance based on clinical judgement; however, the benefit of this strategy has not been proven. Rechallenge protocols that are frequently recommended for reintroduction of TB drugs after an AE are shown in Table 5.

Some authors propose desensitisation therapy for TB drugs after HSR using a carefully structured dosing administration schedule that starts with a very low dose of each drug under close clinical supervision (Supplementary Data).^30^,^33^ The aim of desensitisation is to alter the immune response to the drug resulting in temporary tolerance, allowing the person to safely receive an uninterrupted course of medication. If therapy is interrupted, the hypersensitivity to the medication returns.^47^ Desensitisation may reacquire a drug option; however, success is not guaranteed. This strategy may be appropriate for immediate allergic reactions (Type 1 hypersensitivity) and non-severe delayed reactions, but clinical utility has not been validated in controlled studies. Desensitisation is not recommended in the case of severe cutaneous adverse reactions (SCAR) such as Stevens-Johnson syndrome, toxic epidermal necrolysis or drug reaction with eosinophilia and systemic symptoms (DRESS) syndrome.^32^,^33^,^48^ In all approaches, the risk of new, and possibly, fatal cutaneous AE must be balanced against the increased mortality risk due to TB treatment interruption.^1^,^32^ The procedure should include counselling of the person and members of their support network and/or caregivers regarding the possible risk of anaphylaxis or other systemic reaction, and for need for strict treatment compliance with continued medication doses as prescribed and without interruption following desensitisation.

## STANDARD 6

### Healthcare workers should be trained on AE including how to counsel people undertaking TB treatment, as well as active AE monitoring and management

Education of HCWs is a crucial component of TB treatment programmes and should include training in effective counselling methods, as well as information regarding treatment initiation, the nature of AE, the importance of adherence, common drug-drug interactions and recommended follow-up strategies and schedules. HCW education should emphasise the importance of a person-centred approach to TB care – the guiding principle for counselling about AE and TB treatment. It should include an explanation of the underlying mechanisms of common and important AE for each TB drug, with information about recommended surveillance for common AE, including investigations. Training on available and accessible tools and resources to support monitoring and management of AE should be provided to help build an effective system for active AE monitoring, reporting and management, and support for those undergoing treatment.

Education for HCW should also cover the principles and components of pharmacovigilance and aDSM to ensure timely and accurate recording and reporting of AE during treatment. Training should emphasise the importance of timely, careful, detailed record of the investigation, management, and follow-up of AE. Education should include the recommended management algorithms that should ensue in the event of a particular reaction or symptoms suggestive of AE (Tables 3–5). During HCW training, the importance of assessing the impact of AE on adherence and the TB treatment course should be emphasized. Education of HCW should also include the importance of communication and collaboration with other colleagues involved in the care of people undergoing treatment for TB such as pharmacists and nursing staff as well as their support networks and caregivers. This is particularly important in treating those with TB who also have comorbidities, which require AE monitoring and collaborative management between HCW with different areas of expertise.

## STANDARD 7

### There should be active AE monitoring and reporting for all new TB drugs and regimens

aDSM and management has been introduced for newly approved drugs such as bedaquiline and delamanid. This provides a system for 1) detection, 2) management, and 3) reporting of presumed or confirmed AE using active and systematic clinical and laboratory assessment of people receiving treatment.^49^–^53^ Not only new TB drugs, but also novel regimens, are captured. By prospectively using agreed monitoring strategies, findings from different countries can be easily interpreted and data aggregated, allowing for a timely international/global response to observed and reported AE.^54^ Findings from the analysis could result in immediate safety warnings or prompt alertness for specific AE. Data from an aDSM database should be combined with data from other sources such as the Eudravigilance and the WHO Global Individual Case Safety Report database (VigiBase).^55^,^56^ Active monitoring for AE (depending on the drugs used) should include visual and hearing assessment, neurological/psychiatric evaluation, renal and hepatic function, electrocardiograph (ECG) and electrolytes, lipase and amylase test, blood count, blood glucose and thyroid test.^52^

Depending on available resources, TB programs can focus on 1) only serious AE (SAE), 2) SAE and specific AE, or 3) (ideally) all AE.^57^,^58^ It is of value to involve national medicines regulatory agencies, as they have a legal responsibility for monitoring the safety of the approved medicines. This also helps with making reporting more efficient as duplication of efforts in reporting AE was considered a major hurdle by HCWs.^59^ When reporting a case, the information outlined in Table 6 should be included; person characteristics, event characteristics, seriousness of the event, actions taken and outcome.^52^

**Table 6:** Items to be included when reporting a serious AE (adapted from^52^)

Item	Details
Person affected	Sex, date of birth/age Pregnancy or breastfeeding
Drug (s)	Name and daily dose Date started and stopped
Event	Date event started and stopped Seriousness of event (death, life-threatening event, hospitalisation or prolongation of hospitalisation, persistent or significant disability, congenital anomaly), other
Action taken	Medicine withdrawn, dose increased, dose reduced, dose not changed, unknown
Outcome of event	Recovered/resolved, recovering/resolving, recovered with sequelae, not recovered/not resolved, died, unknown

## STANDARD 8

### Knowledge gaps and signals identified from active AE monitoring should be systematically addressed through clinical research

Many AE identified by HCW are not reported to health authorities at local, national or global level. This underreporting may be due to lack of awareness, administrative burden (need to report to the country and to the aDSM system and to the drug manufacturer with different forms and multiple steps), confidentiality issues, the involvement of different sectors (e.g., public and private, prisons) and the fear of blame.^49^,^60^ An important issue that needs to be addressed in the classification of AE is the attribution of an AE to an individual drug (for establishing causal associations between drugs and AE, see Standard 3). For optimisation of these causal attributions, implementation of standard operating procedures to monitor AE, drug exposure and concomitant drugs need to be carried out and clinical and operational research undertaken. Note should be made of type B reactions, which could be causal, but are not related to dose (e.g., allergic reaction). To assess the relationship between AE and drug exposure (dosage and frequency of administration) pharmacokinetic analysis of the TB drugs may be carried out. For establishing pharmacokinetic exposure parameters, such as ‘area under the curve’ (AUC) values that predict toxicity, classification and regression tree analysis can be used.^61^ Variables related to concomitant medications, which could affect drug exposure, should be recorded.^14^ Because TB drugs are used for prolonged duration and in combination, the cumulative toxicity of a drug should be investigated as well.

## CONCLUSION

Early identification and management of AE during treatment for TB is critical to optimise successful treatment completion. There remains great uncertainty regarding AE risk prior to treatment, and studies into genomic and other predictors should be investigated for developing tailored treatment and monitoring strategies. Programmatic approaches to safe reintroduction of medication after AE should be prioritised, as should evidence-based, person-centred strategies for supporting people with TB through AE safely, and optimal counselling strategies for education regarding AE and reporting.

## References

[1] Nahid P (2016). Official American Thoracic Society/Centers for Disease Control and Prevention/Infectious Diseases Society of America Clinical Practice Guidelines: treatment of drug-susceptible tuberculosis. Clin Infect Dis.

[2] World Health Organisation. (2020). Consolidated guidelines on tuberculosis: Module 4: Treatment - Drug-resistant tuberculosis treatment.

[3] World Health Organisation (2022). Consolidated guidelines on tuberculosis: Module 4: Treatment - Drug-susceptible tuberculosis treatment.

[4] Pradipta IS (2018). Risk factors of multidrug-resistant tuberculosis: a global systematic review and meta-analysis. J Infect.

[5] Awofeso N (2008). Anti-tuberculosis medication side-effects constitute major factor for poor adherence to tuberculosis treatment. Bull World Health Organ.

[6] Thamineni R (2022). Level of adherence to anti-tubercular treatment among drug-sensitive tuberculosis patients on a newly introduced daily dose regimen in South India: a cross-sectional study. Trop Med Int Health.

[7] Oh AL (2023). Prevalence and predictive factors of tuberculosis treatment interruption in the Asia region: a systematic review and meta-analysis. BMJ Glob Health.

[8] Ho YH (2023). Characteristics of patients with tuberculosis and the associated factors with TB-related mortality in a rural setting in Sarawak, Malaysia: a single-centre study. Med J Malaysia.

[9] Sant’Anna FM (2022). Adverse drug reactions related to treatment of drug-susceptible tuberculosis in Brazil: a prospective cohort study. Front Trop Dis.

[10] Arriaga MB (2019). Impact of the change in the antitubercular regimen from three to four drugs on cure and frequency of adverse reactions in tuberculosis patients from Brazil: A retrospective cohort study. PLoS One.

[11] Prasad R, Singh A, Gupta N (2021). Adverse drug reactions with first-line and second-line drugs in treatment of tuberculosis. Ann Natl Acad Med Sci.

[12] Wu S (2016). Adverse Events Associated With the Treatment of Multidrug-Resistant Tuberculosis: A Systematic Review and Meta-analysis. Am J Ther..

[13] Nahid P (2016). Executive Summary: Official American Thoracic Society/Centers for Disease Control and Prevention/Infectious Diseases Society of America Clinical Practice Guidelines: treatment of drug-susceptible tuberculosis. Clin Infect Dis.

[14] Alffenaar JWC (2022). Clinical standards for the dosing and management of TB drugs. Int J Tuberc Lung Dis.

[15] Akkerman OW (2022). Clinical standards for drug-susceptible pulmonary TB. Int J Tuberc Lung Dis.

[16] Foster I (2022). The role of counselling in tuberculosis diagnostic evaluation and contact tracing: scoping review and stakeholder consultation of knowledge and research gaps. BMC Public Health.

[17] Migliori GB (2022). Clinical standards for the diagnosis, treatment and prevention of TB infection. Int J Tuberc Lung Dis.

[18] World Health Organisation (2017). Ethics guidance for the implementation of the End TB strategy.

[19] Centers for Disease Control and Prevention (2022). What is health literacy.

[20] Snider DE, Farer LS (1977). Rifampin and red urine. JAMA.

[21] Explain TB (2023). Information on tuberculosis in over 30 languages. https://www.explaintb.org/contact/en.

[22] Richardson M (2019). NAT2 variants and toxicity related to anti-tuberculosis agents: a systematic review and meta-analysis. Int J Tuberc Lung Dis.

[23] Azuma J (2013). *NAT2* genotype guided regimen reduces isoniazid-induced liver injury and early treatment failure in the 6-month four-drug standard treatment of tuberculosis: a randomized controlled trial for pharmacogenetics-based therapy. Eur J Clin Pharmacol.

[24] Huang D (2023). Time of liver function abnormal identification on prediction of the risk of anti-tuberculosis-induced liver injury. J Clin Transl Hepatol.

[25] Patterson B (2021). Predicting drug-induced liver injury from anti-tuberculous medications by early monitoring of liver tests. J Infect.

[26] Steele MA (1991). Toxic hepatitis with isoniazid and rifampin. A meta-analysis. Chest.

[27] Saukkonen JJ (2006). An official ATS statement: hepatotoxicity of antituberculosis therapy. Am J Respir Crit Care Med.

[28] Naranjo CA (1981). A method for estimating the probability of adverse drug reactions. Clin Pharmacol Ther.

[29] Mehta YS (1996). Rifampicin-induced immune thrombocytopenia. Tubercle Lung Dis.

[30] Sharma RK (2020). Spectrum of cutaneous adverse drug reactions to anti-tubercular drugs and safe therapy after re-challenge - a retrospective study. Indian Dermatol Online J.

[31] Peter JLR, Shear NH, Dodiuk-Gad RP (2018). Cutaneous adverse drug reactions from antituberculosis treatment.

[32] Lehloenya RJ, Dheda K (2012). Cutaneous adverse drug reactions to anti-tuberculosis drugs: state of the art and into the future. Expert Rev Anti Infect Ther.

[33] Bermingham WH (2022). Practical management of suspected hypersensitivity reactions to anti-tuberculosis drugs. Clin Exp Allergy.

[34] Mayorga C (2016). In vitro tests for drug hypersensitivity reactions: an ENDA/EAACI Drug Allergy Interest Group position paper. Allergy.

[35] Bolhuis MS (2019). Treatment of multidrug-resistant tuberculosis using therapeutic drug monitoring: first experiences with sub-300 mg linezolid dosages using in-house made capsules. Eur Respir J.

[36] Migliori GB (2021). Clinical standards for the assessment, management and rehabilitation of post-TB lung disease. Int J Tuberc Lung Dis.

[37] Horter S (2021). Person-centred care in TB. Int J Tuberc Lung Dis.

[38] Conradie F (2022). Bedaquiline-pretomanid-linezolid regimens for drug-resistant tuberculosis. N Engl J Med.

[39] Imperial MZ (2022). Proposed linezolid dosing strategies to minimize adverse events for treatment of extensively drug-resistant tuberculosis. Clin Infect Dis.

[40] Nyang’wa BT (2022). A 24-week, all-oral regimen for rifampin-resistant tuberculosis. N Engl J Med.

[41] National Institute for Health and Care Excellence (2019). Tuberculosis.

[42] Chang KC (2008). Hepatotoxicity of pyrazinamide: cohort and case-control analyses. Am J Respir Crit Care Med.

[43] Kwon BS (2020). The high incidence of severe adverse events due to pyrazinamide in elderly patients with tuberculosis. PLoS One.

[44] Sharma SK (2010). Safety of 3 different reintroduction regimens of antituberculosis drugs after development of antituberculosis treatment-induced hepatotoxicity. Clin Infect Dis.

[45] Saukkonen J (2010). Challenges in reintroducing tuberculosis medications after hepatotoxicity. Clin Infect Dis.

[46] Moosa MS (2022). Rechallenge after anti-tuberculosis drug-induced liver injury in a high HIV prevalence cohort. South Afr J HIV Med.

[47] Cernadas JR (2010). General considerations on rapid desensitization for drug hypersensitivity - a consensus statement. Allergy.

[48] Aberer W (2003). Drug provocation testing in the diagnosis of drug hypersensitivity reactions: general considerations. Allergy.

[49] Borisov S (2019). Surveillance of adverse events in the treatment of drug-resistant tuberculosis: first global report. Eur Respir J.

[50] Borisov SE (2017). Effectiveness and safety of bedaquiline-containing regimens in the treatment of MDR- and XDR-TB: a multicentre study. Eur Respir J.

[51] Koirala S (2021). Outcome of treatment of MDR-TB or drug-resistant patients treated with bedaquiline and delamanid: results from a large global cohort. Pulmonology.

[52] World Health Organisation (2015). Active tuberculosis drug-safety monitoring and management (aDSM). Framework for implementation.

[53] Eurosurveillance (2016). WHO publishes an implementation framework on active tuberculosis drug-safety monitoring and management (ADSM). [Editorial] Euro Surveill.

[54] Halleux CM (2018). The World Health Organization global aDSM database: generating evidence on the safety of new treatment regimens for drug-resistant tuberculosis. Eur Respir J.

[55] European Medicines Agency (2022). Eudravigilance. https://www.ema.europa.eu/en/human-regulatory/research-development/pharmacovigilance/eudravigilance.

[56] VigiBase Uppsala Monitoring Centre. https://who-umc.org/vigibase/.

[57] Lachenal N (2020). Setting up pharmacovigilance based on available endTB Project data for bedaquiline. Int J Tuberc Lung Dis.

[58] Tiemersma E (2019). Integration of drug safety monitoring in tuberculosis treatment programmes: country experiences. Eur Respir Rev.

[59] Tiemersma EW (2021). Baseline assessment of pharmacovigilance activities in four sub-Saharan African countries: a perspective on tuberculosis. BMC Health Serv Res.

[60] Akkerman O (2019). Surveillance of adverse events in the treatment of drug-resistant tuberculosis: a global feasibility study. Int J Infect Dis.

[61] Zheng X (2021). Drug exposure and minimum inhibitory concentration predict pulmonary tuberculosis treatment response. Clin Infect Dis.

[62] European Association for the Study of the Liver (2019). Electronic address eee, et al. EASL Clinical Practice Guidelines: drug-induced liver injury. J Hepatol.

[63] Lucena MI (2020). Drug-induced liver injury in older people. Lancet Gastroenterol Hepatol.

[64] Wilke RA (2007). Identifying genetic risk factors for serious adverse drug reactions: current progress and challenges. Nat Rev Drug Discov.

[65] Breen RA (2006). Adverse events and treatment interruption in tuberculosis patients with and without HIV co-infection. Thorax.

[66] Araujo-Mariz C (2016). Hepatotoxicity during treatment for tuberculosis in people living with HIV/AIDS. PLoS One.

[67] Naidoo K (2020). High rates of drug-induced liver injury in people living with HIV coinfected with tuberculosis (TB) irrespective of antiretroviral therapy timing during antituberculosis treatment: results from the starting antiretroviral therapy at three points in TB trial. Clin Infect Dis.

[68] Nunn AJ (2008). Role of co-trimoxazole prophylaxis in reducing mortality in HIV infected adults being treated for tuberculosis: randomised clinical trial. BMJ.

[69] Thee S (2011). Pharmacokinetics of isoniazid, rifampin, and pyrazinamide in children younger than two years of age with tuberculosis: evidence for implementation of revised World Health Organization recommendations. Antimicrob Agents Chemother.

[70] Chou C (2022). Risk of drug-induced liver injury in chronic hepatitis B and tuberculosis co-infection: A systematic review and meta-analysis. J Viral Hepat.

[71] Lomtadze N (2013). Hepatitis C virus co-infection increases the risk of anti-tuberculosis drug-induced hepatotoxicity among patients with pulmonary tuberculosis. PLoS One.

[72] Wang N (2022). Incidence and temporal trend of antituberculosis drug-induced liver injury: a systematic review and meta-analysis. J Trop Med.

[73] Ngoc NB (2021). Active surveillance for adverse events in patients on longer treatment regimens for multidrug-resistant tuberculosis in Viet Nam. PLoS One.

[74] Khan FU (2022). Assessment of adverse drug events, their risk factors, and management among patients treated for multidrug-resistant tb: a prospective cohort study from Pakistan. Front Pharmacol.

[75] Choi H (2022). Incidence and outcomes of adverse drug reactions to first-line anti-tuberculosis drugs and their effects on the quality of life: a multicenter prospective cohort study. Pharmacoepidemiol Drug Saf.

[76] Siddiqui AN (2016). Effect of diabetes mellitus on tuberculosis treatment outcome and adverse reactions in patients receiving directly observed treatment strategy in India: a prospective study. Biomed Res Int.

[77] Riza AL (2014). Clinical management of concurrent diabetes and tuberculosis and the implications for patient services. Lancet Diabetes Endocrinol.

[78] Munoz-Torrico M (2017). Diabetes is associated with severe adverse events in multidrug-resistant tuberculosis. Arch Bronconeumol.

[79] Kanaujia V (2019). Ethambutol-induced optic neuropathy in renal disorder: a clinico-electrophysiological study. Can J Ophthalmol.

[80] Gupta A (2019). Isoniazid Preventive Therapy in HIV-Infected Pregnant and Postpartum Women. N Engl J Med.

[81] Shakya R (2004). Incidence of hepatotoxicity due to antitubercular medicines and assessment of risk factors. Ann Pharmacother.

[82] Warmelink I (2011). Weight loss during tuberculosis treatment is an important risk factor for drug-induced hepatotoxicity. Br J Nutr.

[83] Podewils LJ (2011). Impact of malnutrition on clinical presentation, clinical course, and mortality in MDR-TB patients. Epidemiol Infect.

[84] Singh J (1995). Antituberculosis treatment-induced hepatotoxicity: role of predictive factors. Postgrad Med J.

[85] Pande JN (1996). Risk factors for hepatotoxicity from antituberculosis drugs: a case-control study. Thorax.

[86] Fernandez-Villar A (2004). The influence of risk factors on the severity of anti-tuberculosis drug-induced hepatotoxicity. Int J Tuberc Lung Dis.

[87] Resende LS, Santos-Neto ET (2015). Risk factors associated with adverse reactions to antituberculosis drugs. J Bras Pneumol.

[88] Chung-Delgado K (2011). Factors associated with anti-tuberculosis medication adverse effects: a case-control study in Lima, Peru. PLoS One.

[89] Sharma SK (2002). Evaluation of clinical and immunogenetic risk factors for the development of hepatotoxicity during antituberculosis treatment. Am J Respir Crit Care Med.

[90] Gafar F (2019). Antituberculosis drug-induced liver injury in children: incidence and risk factors during the two-month intensive phase of therapy. Pediatr Infect Dis J.

[91] Lorent N (2011). Incidence and risk factors of serious adverse events during antituberculous treatment in Rwanda: a prospective cohort study. PLoS One.

[92] Lan Z (2020). Drug-associated adverse events in the treatment of multidrug-resistant tuberculosis: an individual patient data meta-analysis. Lancet Respir Med.

[93] Schnippel K (2017). Adverse drug reactions during drug-resistant TB treatment in high HIV prevalence settings: a systematic review and meta-analysis. J Antimicrob Chemother.

[94] Huangfu P (2019). The effects of diabetes on tuberculosis treatment outcomes: an updated systematic review and meta-analysis. Int J Tuberc Lung Dis.

[95] Huangfu P (2019). Point of care HbA(1c) level for diabetes mellitus management and its accuracy among tuberculosis patients: a study in four countries. Int J Tuberc Lung Dis.

[96] Jeon CY (2012). Managing tuberculosis in patients with diabetes mellitus: why we care and what we know. Expert Rev Anti Infect Ther.

[97] Mukonzo J (2019). Potential drug-drug interactions between antiretroviral therapy and treatment regimens for multi-drug resistant tuberculosis: implications for HIV care of MDR-TB co-infected individuals. Int J Infect Dis.

[98] Sousa M (2008). Pharmacokinetics and pharmacodynamics of drug interactions involving rifampicin, rifabutin and anti-malarial drugs. J Antimicrob Chemother.

[99] Duvenhage J (2022). Hepatocellular injury in children treated for rifampicin-resistant tuberculosis: incidence, etiology and outcome. Pediatr Infect Dis J..

[100] Ozgur OK (2018). Validity and acceptance of color vision testing on smartphones. J Neuroophthalmol.

[101] Beidas RS (2015). Free, brief, and validated: Standardized instruments for low-resource mental health settings. Cogn Behav Pract.

[102] Senousy BE (2010). Hepatotoxic effects of therapies for tuberculosis. Nat Rev Gastroenterol Hepatol.

[103] Court R (2021). Neuropsychiatric toxicity and cycloserine concentrations during treatment for multidrug-resistant tuberculosis. Int J Infect Dis.

[104] Ata F (2022). Rifampin-induced acute kidney injury and hemolysis: a case report and literature review of a rare condition. Clin Case Rep.

[105] Muthukumar T (2002). Acute renal failure due to rifampicin: a study of 25 patients. Am J Kidney Dis.

[106] Grilo Novais A (2021). Rifampicin-induced nephrotoxicity in a tuberculosis patient: treatment dilemma. Eur J Case Rep Intern Med.

