# Supplementary material for: Clinical standards for the management of adverse effects during treatment for TB
Source: Int J Tuberc Lung Dis. 2023 Jul 1;27(7):506–19. doi: 10.5588/ijtld.23.0078 (PMC10321364; doi:10.5588/ijtld.23.0078)
Supplement: Supplementary file 1 [file iutld_ijtld_23.0078_supplementarydata1.pdf]

http://dx.doi.org/10.5588/ijtld.23.0078

Clinical standards for the management of adverse effects during treatment for TB

SUPPLEMENTARY DATA

Supplementary Data 1

Rapid desensitization protocols

Rapid desensitization involves a structured, graded administration of a drug under close clinical supervision and is primarily used in people with a history of an immediate allergy (type-1 HSR) reaction.
There are no randomised controlled trials to validate this approach and most evidence comes from published case series and case reports. Where available, specialist input should be sought. Further information on appropriate use of desensitization can be found in an international consensus document[48].

Indications:
•History of or presumed immediate allergic reaction (type I hypersensitivity)
•Uncertain history of an allergic reaction
•History of mild non-immediate HSRs (type IV HSR) (usually benign cutaneous)

Governance:
•The person (and/or support-person/caregiver) should be counselled regarding an unquantifiable possible risk of systemic reaction or anaphylaxis, document in notes and obtain written informed consent. Counsel the person (and/or support-person/caregiver) regarding the need for strict treatment compliance following desensitization. Provide details of where they can access clinical advice in the event of missed doses.
•The procedure should be undertaken in an intensive care setting or medical ward with a 1:1 supervision of senior staff nurse with the availability of a clinician on-site competent in treating anaphylaxis and with immediate access to cardiopulmonary resuscitation. Where available, an intensive care specialist or critical care/intensive care outreach team may be consulted in planning desensitization.
•Involve a clinical pharmacist to prepare drug dilutions and advise nursing staff regarding the safe execution of the protocol. Such procedures commonly utilise licenced preparations being delivered in an unlicensed form, and therefore appropriate local authorisation for this should be sought.

Safety measures:
Perform a risk-benefit analysis.
1.Optimise cardio-respiratory status (e.g., asthma, COPD, heart function).
The risk of inducing anaphylaxis needs to be considered in the context of the person's current/pre-existing clinical state.
2.Discontinue beta-blockers if deemed safe (otherwise keep glucagon ready for the treatment of refractory anaphylaxis).

3.Antihistamine/s and other drugs with antihistamine property are preferably discontinued. Drug half-life should be considered with appropriate washout period. However, where this is not possible, note that such medicines may mask early signs of an HSR during desensitization.

Procedure:
1.Peripheral vein cannulation for IV access in the context of desensitization for type I HSRs.
2.Check baseline vital parameters including pulse/heart rate, blood pressure (BP) and peak expiratory flow rate (PEFR)or ventilator pressures for people on life support machines
3.Carefully monitor the person for symptoms of an allergic reaction including rash, swelling (angioedema), cough, rhinitis, respiratory distress, or hypotension.
4.Check vital parameters prior to each step. If there are concerns regarding an allergic reaction, stop the procedure, and call for immediate medical attention.
5.Allergic reaction: Treat promptly. After stabilisation consider dose adjustments. This may require going back few steps in the protocol and/or adding an intermediate step.
6.Observe the person for 1 hour after the final step. If the therapeutic dose is well tolerated, regular administration of this dose should be carried out until the treatment course is completed.
7. Note that immunological tolerance is maintained only for the duration of treatment and tolerance status is lost within a few days following discontinuation of therapy.

AN EXAMPLE OF RAPID ORAL RIFAMPICIN DESENSITIZATION (adapted from [63, 64])

**Solution**	**Volume (mL) to reconstitute**	**Concentration (mg/mL)**	**Total volume required (mL)**
A	10	0.001	0.2
B	10	0.01	2.2
C	30	0.1	2
D	10	6	5
E	15	60	9.7

**Step**	**Solution**	**Volume (mL)**	**Dose administered (mg)**	**Cumulative dose (mg)**	**Time, (minutes following first dose)**
1	A	0.2	0.0002	0.0002	0
2	B	0.2	0.002	0.002	30
3	B	2	0.02	0.02	60
4	C	2	0.2	0.2	90
5	D	0.3	1.8	2.0	120
6	D	0.7	4.2	6.2	150
7	D	1.3	7.8	14.0	180
8	D	2.7	16.2	30.2	210
9	E	0.5	30	60.2	240
10	E	0.8	48	108.2	270
11	E	1.7	102.	210.2	300
12	E	2.5	150	360.2	330
13	E	4.2	252	612.2	360

ISONIAZID DESENSITIZATION (from [65])

It is important to note that this protocol results in a cumulative dose of 560mg that is more than the typical isoniazid daily therapeutic dose for an adult person.

**Step**	**Dose administered (mg)**	**Cumulative dose (mg)**	**Time, (minutes following first dose)**
1	0.1	0.1	0
2	0.5	0.6	30
3	1	1.6	60
4	2	3.6	90
5	4	7.6	120
6	8	15.6	150
7	16	31.6	180
8	32	63.6	210
9	50	113.6	240
10	100	213.6	270
11	150	363.6	300
12	200	563.6	330
